# Roles of Eph-Ephrin Signaling in the Eye Lens Cataractogenesis, Biomechanics, and Homeostasis

**DOI:** 10.3389/fcell.2022.852236

**Published:** 2022-02-28

**Authors:** Subashree Murugan, Catherine Cheng

**Affiliations:** Indiana University, School of Optometry and Vision Science Program, Bloomington, IN, United States

**Keywords:** EphA2, ephrin-A5, fiber cell, epithelial cell, suture

## Abstract

The eye lens is responsible for fine focusing of light onto the retina, and its function relies on tissue transparency and biomechanical properties. Recent studies have demonstrated the importance of Eph-ephrin signaling for the maintenance of life-long lens homeostasis. The binding of Eph receptor tyrosine kinases to ephrin ligands leads to a bidirectional signaling pathway that controls many cellular processes. In particular, dysfunction of the receptor EphA2 or the ligand ephrin-A5 lead to a variety of congenital and age-related cataracts, defined as any opacity in the lens, in human patients. In addition, a wealth of animal studies reveal the unique and overlapping functions of EphA2 and ephrin-A5 in lens cell shape, cell organization and patterning, and overall tissue optical and biomechanical properties. Significant differences in lens phenotypes of mouse models with disrupted EphA2 or ephrin-A5 signaling indicate that genetic modifiers likely affect cataract phenotypes and progression, suggesting a possible reason for the variability of human cataracts due to Eph-ephrin dysfunction. This review summarizes the roles of EphA2 and ephrin-A5 in the lens and suggests future avenues of study.

## Introduction

Eph-ephrin signaling plays an important role in development, homeostasis, and disease in humans (Henkemeyer et al., 1994; Holmberg et al., 2000; Clevers and Batlle, 2006; Zhao et al., 2006) and other organisms (Park et al., 2004; Picco et al., 2007; Lisabeth et al., 2013). This signaling pathway can act bidirectionally to initiate canonical signaling through kinase activity if receptor-ligand interaction occurs *in trans* on neighboring cells or can bring about non-canonical signaling if the receptors or ligands signal *in cis* within a single cell (Liang et al., 2019). Several organ systems and diseases influenced by this canonical or non-canonical pathway have been reviewed in detail previously (O'Leary and Wilkinson, 1999; Pasquale, 2004; Himanen et al., 2007; Pasquale, 2008, 2010; Lisabeth et al., 2013; Barquilla and Pasquale, 2015; Kania and Klein, 2016; Darling and Lamb, 2019; Defourny, 2019; Kaczmarek et al., 2021). Recently, disruption of Eph-ephrin signaling in human patients has been associated with congenital and age-related cataracts, defined as any opacity in the transparent eye lens (Shiels et al., 2008; Jun et al., 2009; Zhang et al., 2009; Kaul et al., 2010; Tan et al., 2011; Sundaresan et al., 2012; Dave et al., 2013; Patel et al., 2017; Berry et al., 2018; Zhai et al., 2019). Mouse models are now being used to understand the mechanism of cataractogenesis (Cooper et al., 2008; Jun et al., 2009; Cheng and Gong, 2011; Shi et al., 2012; Cheng et al., 2013; Son et al., 2013; Biswas et al., 2016; Cheng et al., 2017; Cheng et al., 2021; Zhou et al., 2021; Cheng et al., 2022). In this review, we highlight the different functions of Eph-ephrin signaling in the lens, describe how genetic background influences cataract phenotypes, as well as provide some insights into future directions and potential therapeutic strategies that can be tested to understand the pathogenesis of age-related cataracts.

### Eph-ephrin Bidirectional Signaling

**FIGURE 1:**
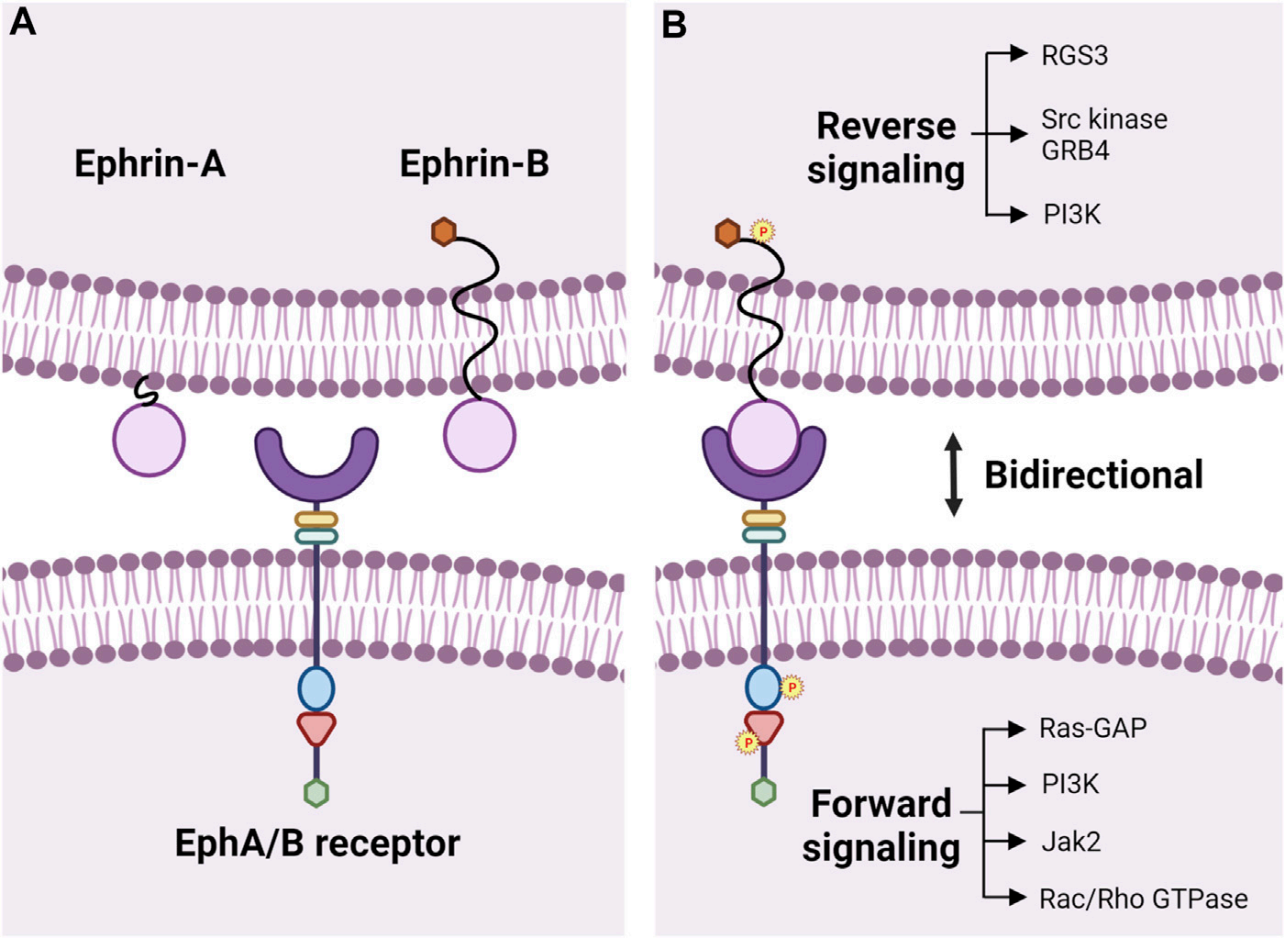
Eph-ephrin bidirectional signaling. **(A)** Transmembrane Eph receptor tyrosine kinases consist of a ligand binding domain (purple semi-circle), cysteine-rich EGF-like motif (yellow rectangle), and fibronectin type III repeats (green rectangle) in the extracellular region and have a tyrosine kinase domain (blue oval), SAM domain (red triangle), and a PDZ-binding motif (green hexagon) intracellularly. The extracellular and intracellular domains are linked by a transmembrane domain. Eph receptors are divided into two classes, EphAs, and EphBs, and bind to membrane-bound ligands called ephrins. Ephrin-As are membrane-anchored via a glycosylphosphatidylinositol (GPI) moiety, and ephrin-Bs have a transmembrane domain with a short cytoplasmic extension containing a PDZ-binding motif (orange hexagon) for autophosphorylation. **(B)** Binding of Ephs to ephrins leads to bidirectional signaling with forward signaling in the Eph-bearing cell and reverse signaling in the ephrin-bearing cell through phosphorylation of tyrosine residues. Downstream activation of various kinases and pathways has been reviewed in detail previously (Pasquale, 2008). Illustration not drawn to scale and created with the aid of BioRender.com.

Erythropoietin-producing hepatocellular carcinoma (Eph) receptors are transmembrane proteins that make up a large subfamily of receptor tyrosine kinases (RTK) (Pasquale, 2010; Pitulescu and Adams, 2010; Darling and Lamb, 2019). Eph receptors interact with cell surface-bound ligands, known as Eph receptor-interacting proteins (ephrins), to mediate many important cellular functions, including cell proliferation (Zhang et al., 2001), migration (Davy and Robbins, 2000), adhesion (Davy et al., 1999; Davy and Robbins, 2000), and repulsion (Holmberg et al., 2000). Human Eph receptors are divided into two subclasses, EphAs (nine members; A1-8, A10) and EphBs (five members; B1-4, B6), based on their sequence similarity and ligand affinity (Pasquale, 2004; 2005; Himanen et al., 2007; Noberini et al., 2012; Lisabeth et al., 2013; Darling and Lamb, 2019). The extracellular region of Eph receptors consists of an ephrin-binding domain, a cysteine-rich EGF-like motif, and 2 fibronectin repeats (type III). The intracellular region of Eph receptors is made up of a tyrosine kinase domain, sterile alpha motif (SAM), and a PDZ-binding motif; the extracellular and intracellular domains are connected through a transmembrane section (Figure 1A) (Davis et al., 2008; Darling and Lamb, 2019). The ligands are categorized into two groups based on their structural differences. Ephrin-As (five members; A1-5) are anchored via a glycosylphosphatidylinositol (GPI) moiety to the membrane, while ephrin-Bs (three members; B1-3) traverse the cell membrane and have a short cytoplasmic extension (Figure 1A) (Kullander and Klein, 2002; Pasquale, 2004, 2005; Darling and Lamb, 2019).

Due to the membrane-bound nature of these receptors and ligands, cell-cell contact and binding between Ephs and ephrins are required to initiate intracellular signals for canonical ligand-mediated signaling, in which dimerization of the Eph receptors is regulated by the clustering of ligand complexes (Poliakov et al., 2004; Liang et al., 2019). Interactions between Eph receptors and ephrin ligands influence several physiological processes during development and aging, like axon guidance (Pasquale et al., 1992; Henkemeyer et al., 1994), tissue patterning (Xu et al., 1995), angiogenesis in developing embryos (Wang et al., 1998), bone homeostasis (Zhao et al., 2006), insulin production (Konstantinova et al., 2007), immune surveillance (Luo et al., 2011; Darling and Lamb, 2019), retinal cell patterning (Frisen et al., 1998; Marler et al., 2008), cochlear development (Defourny, 2019), actin cytoskeleton regulation (Carter et al., 2002; Yang et al., 2006; Cheng et al., 2013), cellular adhesion through intercellular junctions (Jorgensen et al., 2009), and cell migration (Matsuoka et al., 2005; Pasquale, 2008). EphA and EphB receptors mainly interact and bind to ephrin-As and ephrin-Bs, respectively (Takemoto et al., 2002; Himanen et al., 2004), and each receptor can interact with multiple ligands, and vice versa (Gale et al., 1996). Cross interactions between EphAs and ephrin-Bs or EphBs and ephrin-As can also occur, though those interactions are relatively less common (Gale et al., 1996; Kullander and Klein, 2002). Upon receptor-ligand binding, the signaling pathway acts bidirectionally to initiate forward signaling through receptor kinase activity and reverse signaling in the ligand-bearing cell (Liang et al., 2019). Forward signaling in Ephs involves phosphorylation of tyrosines in a juxtamembrane location located N-terminal to the tryosine kinase domain and within the activation loop of the tyrosine kinase domain (Wiesner et al., 2006; Fang et al., 2008; Balasubramaniam et al., 2011; Taylor et al., 2017; Liang et al., 2019). Reverse signaling in ephrin-As usually requires recruitment of other kinases to the cell membrane (e.g. Fyn, a member of the Src kinase family), and activation of ephrin-Bs occurs through the phosphorylation of the tyrosinses in the cytoplasmic tail by Src family kinases (Pasquale, 2008; Lisabeth et al., 2013; Taylor et al., 2017; Wu et al., 2019). Although ephrin-As do not have a cytoplasmic tail, they can still activate intracellular signals, *in cis*, within the cell and *in trans*, on neighboring cells (Lisabeth et al., 2013). Non-canonical signaling that is independent of ligand or receptor binding can also occur. Ephrin-independent non-canonical EphA2 signaling is a hallmark of cancers where the receptor is upregulated, accompanied by low expression of ephrin-As or dysfunction of forward signaling in ephrin-A-bearing cells (Gopal et al., 2011; Stahl et al., 2011; Lisabeth et al., 2013). Non-canonical ligand-independent EphA2 signaling depends on phosphorylation of S897 in linker segment connecting the tyrosine kinase and SAM domains by Akt, Rsk, or PKA, leading to increased cell invasion (Miao et al., 2009; Zhou et al., 2015; Barquilla et al., 2016). The ephrin-B1 ligand can induce a cellular response by transducing signals independently, without being activated by any Eph receptors, through phosphorylation by fibroblast growth factor receptors (FGFR) (Lee et al., 2009).

Eph-ephrin signaling initates widespread signal cascades during development, growth, and disease in various tissues and organ systems through cell-cell interactions. These signaling pathways have been reviewed in detail previously (Pasquale, 2008), and we provide a brief overview here (Figure 1B). Crosstalk between integrins and Eph-ephrin signaling results in cell-cell adhesion (Davy and Robbins, 2000; Gu and Park, 2001). These two pathways meet at the level of cytoplasmic kinases including PI3K, MAPK or small GTPases, such as Rho, Rac or Ras (Figure 1B) (Arvanitis and Davy, 2008). E-cadherin can play a direct role by inhibiting phosphorylation of EphA2 leading to cell adhesion or have an indirect function by stabilizing cell-cell contacts to promote interactions between ephrins and Ephs, including EphB/ephrin-B binding, to promote adherens junction formation (Zantek et al., 1999; Ireton and Chen, 2005; Noren and Pasquale, 2007). Downstream effectors of Eph-ephrin signaling, like Rac/Rho GTPases, are responsible for the cytoskeletal organization and cell-cell interactions involving cell shape, adhesion, and migration (Lisabeth et al., 2013). PI3K-Akt/PKB and Ras/MAPK signaling have been reported to be influenced by EphA2 during cell migration and cell proliferation, respectively (Jiang et al., 2015; Liang et al., 2019). Gap junctions and connexins are involved in embryo patterning and organogenesis, and gap junction communication can be inhibited by the Eph-ephrin signaling (Mellitzer et al., 1999; Arvanitis and Davy, 2008). Claudins interact with EphA2 or ephrin-B1 to control intercellular permeabilization and cell adhesion (Arvanitis and Davy, 2008).

### Eye Lens Pathology and Eph-ephrin Signaling in Human Lenses

**FIGURE 2:**
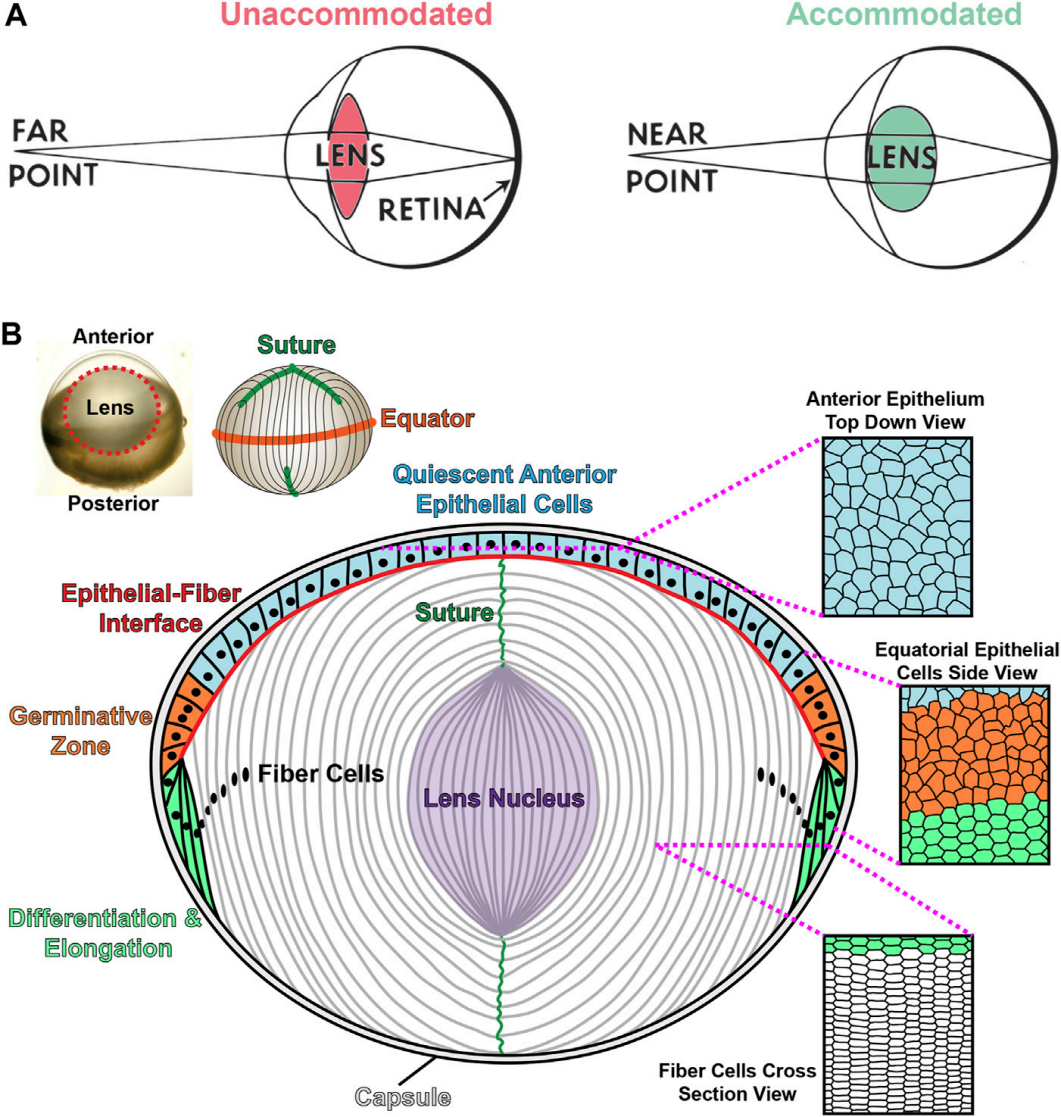
Lens accommodation and anatomy. **(A)** The lens changes shape to fine focus light coming from sources at various distances onto the retina. When viewing objects that are far away, the lens is unaccommodated and relatively flat (left). During accommodation, the lens becomes more spherical to focus near objects (right). Adapted from an open-source Pearson Scott Foster illustration (not drawn to scale). **(B)** An illustration (not drawn to scale) depicting a longitudinal (anterior-posterior) section of the lens with a monolayer of epithelial cells on the anterior hemisphere (colored cells) and a bulk mass of elongated lens fibers (white cells). Lens fibers extend from the anterior to posterior poles. The lens capsule, a thin basement membrane, encapsulates the entire tissue. Anterior epithelial cells (blue) are cobblestone in shape and quiescent. These cells normally do not proliferate. Equatorial epithelial cells (orange) in the germinative zone proliferate, migrate and differentiate into new layers of lens fibers. During migration and differentiation, equatorial epithelial cells transform from randomly organized cells (orange) into highly organized hexagonal cells arranged into neat rows (green). Lifelong lens growth depends on the addition of new fiber cells in concentric shells at the periphery of the lens. Lens fibers retain the organized hexagonal rows as seen in the cross-section view. Newly formed fibers elongate toward the anterior and posterior poles, migrating along the apical surface of epithelial cells or the posterior capsule, respectively. Fully elongated fibers at the anterior and posterior poles will detach from the epithelial cells or lens capsule and contact the elongating fiber from the opposing sides forming the Y-suture. Fiber cell maturation eliminates light-scattering cell organelles in the inner fiber cells, and the lens nucleus, or the central core of the tissue, is composed of tightly compacted fiber cells in the middle of the lens (purple). Modified from (Cheng et al., 2019).

The eye has two main refractive tissues, the cornea and the lens. Although the cornea contributes 2/3 of the focusing power of the eye, the lens is responsible for the fine focusing component of vision. The lens is a transparent, ellipsoid organ in the anterior chamber that changes shape to focus light from objects that are far or near (Lovicu and Robinson, 2004). During accommodation, the lens becomes more convex to focus light from near objects clearly onto the retina (Figure 2A). With age, the lens loses its accommodative function, resulting in presbyopia and the need for reading glasses (Lovicu and Robinson, 2004; Michael and Bron, 2011). The increasing stiffness of the aging lens has been postulated to be a cause for presbyopia (Heys et al., 2004; Heys et al., 2007; Weeber et al., 2007). In addition to its biomechanical properties, the transparency of the lens is essential to its function. Cataracts are the leading cause of blindness worldwide (World Health Organization, 2019). There are several risk factors associated with cataract formation, such as exposure to UV radiation, the effects of reactive oxygen species, nutritional deficits, and the influence of genetic mutations (Shiels and Hejtmancik, 2017; Sella and Afshari, 2019; Uwineza et al., 2019). However, little is known about the cellular and molecular mechanisms for age-related cataracts. Currently, surgery is the only option to remove cataracts, and there are no treatments to prevent or delay cataracts.

Recent reports have linked dysfunction of Eph-ephrin signaling to congenital and age-related cataracts in human patients. Mutations in the *EPHA2* gene can cause a variety of congenital (Zhang et al., 2009; Kaul et al., 2010; Park et al., 2012; Dave et al., 2013; Li et al., 2016; Berry et al., 2018; Zhai et al., 2019) and age-related (Jun et al., 2009; Tan et al., 2011; Sundaresan et al., 2012; Lin et al., 2014) cataracts (Table 1). Non-synonymous single nucleotide polymorphisms (nsSNPs) in the *EFNA5* gene, which encodes the ephrin-A5 protein, have also been reported to cause age-related cataracts in humans (Table 1) (Lin et al., 2014). nsSNPs are missense or nonsense mutations resulting from the substitution of a single nucleotide leading to one amino acid change in a protein sequence that could potentially, but not necessarily, affect the protein structure, folding, interactions, and/or functions (Yates and Sternberg, 2013; Zaharan et al., 2018). In addition to nsSNPs in the coding region of genes, there are also non-coding SNP. SNP rs6603883, which is in the promoter region of *EPHA2* within the PAX2-binding motif, has been reported to affect EphA2 protein levels. This downregulation of EphA2 levels alters the downstream MAPK/AKT pathway and affects other extracellular matrix (ECM) and cytoskeletal genes to cause cataracts (Ma et al., 2017). Studies of *EPHA2* and *EFNA5* mutations have been carried out in diverse populations, including American, Indian, Pakistani, Chinese, British, and Australian families (Shiels et al., 2008; Zhang et al., 2009; Sundaresan et al., 2012). The most common *EPHA2* mutations occur in the tyrosine kinase domain, which affects adherens junctions (Jun et al., 2009; Kaul et al., 2010; Patel et al., 2017; Zhai et al., 2019), or in the SAM domain, which results in structural disruption of the EphA2 receptor (Shiels et al., 2008; Zhang et al., 2009; Dave et al., 2013; Shentu et al., 2013). Several nsSNPs in *EPHA2* affect the stability and translational regulation of the protein (Lin et al., 2014; Li et al., 2016; Li et al., 2021) and have been associated with congenital and age-related cataracts in humans (Jun et al., 2009; Kaul et al., 2010; Dave et al., 2013; Zhai et al., 2019; Li et al., 2021). The mechanisms for cataractogenesis in human patients with these mutations remain unclear and require further study. Hence, several groups are working on knockout or mutant mouse models to dissect the roles of Eph-ephrin signaling in cataractogenesis and lens homeostasis.

**TABLE 1:** *EPHA2* and *EFNA5* cataract-causing mutations in humans.

*EPHA2* mutation (location)	Population	Phenotype	Potential cause	References
Juxtamembrane domain mutation (p.Pro548Leu); SAM domain variants (p.Asp942fs + Cys71); (p.Ala959Thr)	South-Eastern Australians - AD	Nuclear, total, subcapsular, and cortical congenital cataract	Affected phosphorylation profile of tyrosine residues	Dave et al. (2013)
Mutation in the tyrosine kinase domain (p.Gly668Asp)	Han Chinese family - AD	Congenital posterior sub-capsular cataract	Destabilization of EphA2, change in amino acid polarity, change in subcellular localization	Zhai et al. (2019)
Kinase domain mutation (p.Gln669His)	Saudi Arabian family - AD	Nuclear, posterior subcapsular infantile cataract	Not known	Patel et al. (2017)
Kinase domain mutation (p.Ala785Thr)	Pakistani family - AR	Autosomal recessive congenital cataracts	Deleterious effect on the protein structure, effect on adherens junction	Kaul et al. (2010)
SAM domain mutation (p.Arg890Cys)	Chinese family - AD	Progressive congenital posterior sub-capsular cataract	Structural alteration of EphA2 protein	Shentu et al. (2013)
SAM domain mutations (p.Thr940Ile); (p.Val972GlyfsX39); (c.2826-9G>A)	Chinese, British, and Australian families - AD	Congenital posterior polar cataract	Defective oligomerization interface, Loss of function due to binding with Low molecular weight protein tyrosine phosphatase (LMW-PTP)	Zhang et al. (2009)
SAM domain mutation (p.Gly948Trp)	American family - AD	Congenital posterior subcapsular cataract	EphA2 receptor dysfunction	Shiels et al. (2008)
Synonymous mutation (p.Lys935); Non-synonymous mutation (p.Glu934Lys)	Han Chinese - Sporadic	Sporadic congenital cataracts (total/cortical cataract)	Not known	Li et al. (2016)
Recurrent splice-site mutation c.2826-9G>A in EPHA2 gene	British family - AD	Congenital posterior nuclear cataracts	Not known	Berry et al. (2018)
Intergenic variant (rs477558 G>A) and regulatory region variant (rs7548209 G>C)	Han Chinese	Age-related cortical cataracts	Not known	Tan et al. (2011)
Intergenic variant (rs477558 G>A) and regulatory region variant (rs7548209 G>C), Intron variant (rs3768293 G>A,C,T)	Han Chinese	Age-related cataracts	Not known	(Huang et al., 2019)
Non-synonymous SNP (rs137853199 C>A)	Han Chinese	Age-related cortical cataracts	Altered protein stability and degradation, and cell mobility	Li et al. (2021)
Non-synonymous SNPs (rs2291806 C>T)	SNP database	Age-related cataracts	Not known	(Masoodi et al., 2012)
3′ EphA2 SNP (rs7543472 C>T)	Indians	Age-related posterior sub-capsular cataracts	Not known	Sundaresan et al. (2012)
3′ EphA2 SNP (rs7543472 C>T)	Indians	Age-related cataracts (nuclear, cortical, posterior-sub-capsular and mixed cataract)	Not known	(Aslam et al., 2020)
Tyrosine kinase domain mutation (c.Arg721Gln); Regulatory region mutation (rs7548209 G>C); Synonymous mutation (rs6678616 C>G/T)	Caucasians	Age-related cortical cataracts	Impaired adherens junction and cellular stress	Jun et al. (2009)
Synonymous polymorphism rs3754334	Meta-analysis (Indian, Chinese and American populations)	Age-related cataracts	Changes in the *EPHA2* protein configuration	(Yang et al., 2013)
Functional non-coding SNP rs6603883 in the promoter region	Americans (Cystinosis samples)	Age-related cataracts	Alterations in the MAPK/ AKT signaling pathways, extracellular matrix and cytoskeletal genes	Ma et al. (2017)

*EFNA5* mutation (Location)	Population	Phenotype	Potential cause	References
Non-Synonymous SNPs (c.668C>T – p.Ala223Val), (c.-27C>G), Synonymous SNP (c.102C>T)	Chinese	Age-related cataracts	Affect translational and post-translational regulation	Lin et al. (2014)

AD, Autosomal dominant; AR, autosomal recessive; SNPs, Single nucleotide polymorphisms; SAM, Sterile-alpha motif.

### Roles of Ephrin-A5 in Maintaining Anterior Epithelial Cells and Fiber Cells

**FIGURE 3:**
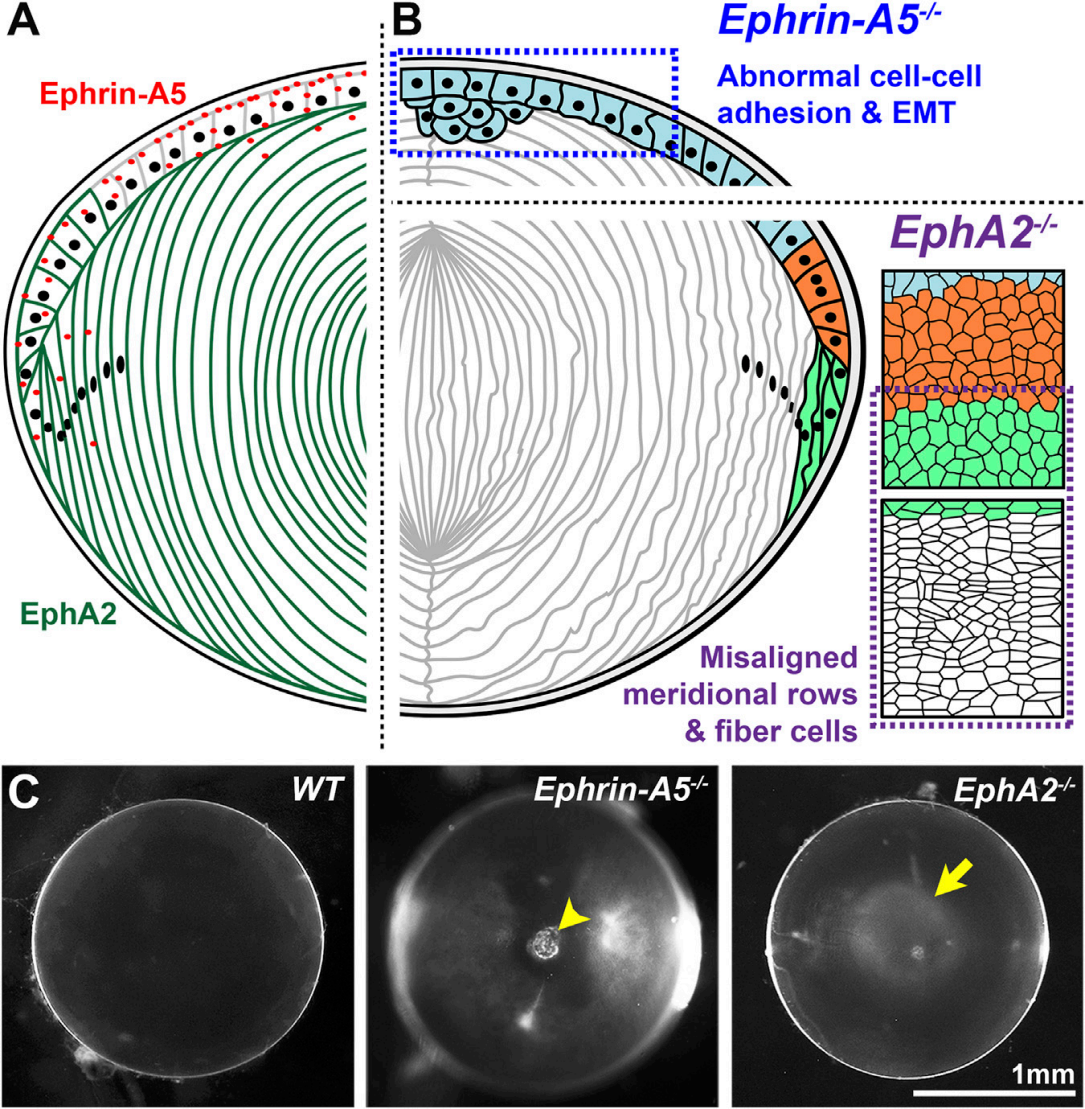
EphA2 and ephrin-A5 in mouse lenses. **(A)** EphA2 (green) is mainly expressed in equatorial epithelial cells and lens fiber cells, while ephrin-A5 (red) is mainly present in anterior epithelial cells with some expression in peripheral fiber cells and in fiber cell tips near the lens suture. **(B)** In *C57BL/6J* genetic background mice, loss of ephrin-A5 leads to abnormal cell-cell adhesion between anterior epithelial cells and epithelial-to-mesenchymal transition (EMT) of these normally quiescent cells. In contrast, disruption of EphA2 in *C57BL/6J* mice leads to disorder of the equatorial epithelial cells, which leads to abnormal lens fiber cell shape. **(C)** The normal wild-type (*WT*) lens is clear on a darkfield background. In contrast, *ephrin-A5* ^*−/−*^ lenses often have anterior cataracts (arrowhead), and *EphA2* ^*−/−*^ lenses often display nuclear cataracts at the center of the lens (arrow). These images are of lenses from three-week-old mice in the *C57BL/6J* genetic background. Modified from (Cheng et al., 2017). Illustrations are not drawn to scale. Scale bar, 1 mm.

The lens, derived from the surface ectoderm (McAvoy, 1978a; McAvoy, 1978b), is an ellipsoidal mass of cells composed of a monolayer of epithelial cells covering the anterior hemisphere and many layers of concentrically organized fiber cells extending from the anterior to posterior poles (Figure 2B) (Lovicu and Robinson, 2004). The entire lens is encapsulated by a basement membrane, known as the lens capsule (Lovicu and Robinson, 2004). The anterior epithelial cells are normally mitotically inactive while epithelial cells in the germinative zone of the equatorial region undergo continuous proliferation, migration, differentiation, and elongation to form new generations of lens fiber cells (Figure 2B) (Piatigorsky, 1981; Kuszak et al., 2004a; Kuszak et al., 2006). Direct immunofluorescence studies showed that the ephrin-A5 protein is detected in anterior epithelial cells, anterior tips of fiber cells and peripheral equatorial fibers in mouse lenses (Figure 3A) (Cheng and Gong, 2011; Cheng et al., 2017; Zhou and Shiels, 2018). Indirect immunofluorescence, using EphA5–alkaline phosphatase affinity probe for ephrin ligand detection, showed a similar epithelial and peripheral fiber staining pattern for ephrin-A5 in the lens (Son et al., 2013).

The lens phenotype for ephrin-A5 knockout (^−/−^ or KO) mice varies greatly depending on genetic background (Table 2) (Cooper et al., 2008; Cheng and Gong, 2011; Son et al., 2013; Biswas et al., 2016; Cheng et al., 2017). *Ephrin-A5* ^*−/−*^ mice in a mixed genetic (*129/Sv* *:C57BL/6*) background have severe and nearly whole cataracts at 6 months of age with posterior capsule rupture, and lenses from younger mice have many cellular abnormalities, including vacuoles and alterations in the fiber cell shape, size, organization, and packing (Cooper et al., 2008; Son et al., 2013; Biswas et al., 2016; Zhou and Shiels, 2018). In *C57BL/6J* background mice, *ephrin-A5* ^*−/−*^ lenses displayed anterior polar cataracts caused by abnormal proliferation of anterior epithelial cells undergoing epithelial-to-mesenchymal (EMT) transition (Figure 3B) (Cheng and Gong, 2011; Cheng et al., 2017). *Ephrin-A5* ^*−/−*^ anterior epithelial cells showed punctate, rather than membrane-localized, β-catenin immunostaining signals along with abnormal E-cadherin staining (Cheng and Gong, 2011). These defects in cell-cell adhesion through adherens junctions likely lead to EMT, and the cluster of abnormal anterior epithelial cells invade the underlying fiber cell layer to cause anterior cataracts in the *ephrin-A5* ^*−/−*^ mice (Figures 3B,C) (Cheng and Gong, 2011). Interestingly, the hexagonal packing of fiber cells in *ephrin-A5* ^*−/−*^ lenses in *C57BL/6J* background mice appears relatively normal (Cheng and Gong, 2011; Cheng et al., 2017; Cheng et al., 2021). Based on these studies, genetic background strongly influences cataract phenotype and severity in *ephrin-A5* ^*−/−*^ mice. While there are anterior epithelial cell defects and cataracts in *C57BL/6J* background *ephrin-A5* ^*−/−*^ lenses, severe fiber cell defects are more obvious in mixed (*129/Sv:C57BL/6*) background KO mice.

**TABLE 2:** EphA2 and ephrin-A5 knockouts and mutations in mice.

*EphA2* ^*−/−*^ genetic background	Knock-out/-in strategy	Phenotype (age)	Cellular changes	Potential cause for cataracts	References
*129/SvJ:C57BL/6J*	Secretory gene trapping (intron 1)	Cortical cataracts progressing to involve the whole lens and lens rupture (not provided)			Jun et al. (2009)
*FVB/NJ*	Secretory gene trapping (between exon 5 and intron 6)	Cortical cataracts (3 months) progressing to involve the whole lens (6 months) and finally lens rupture (8 months)	Clusters of cortical vacuoles (1 month), upregulation of Hsp25 protein	Cellular stress and protein misfolding	Jun et al. (2009)
*Mixed FVB:C57BL6J*	Secretory gene trapping (between exon 5 and intron 6)	Mild anterior cortical lens opacity (11 weeks); severe anterior cortical opacities (18 weeks)			Dave et al. (2021)
*C57BL/6J*	Secretory gene trapping (between exon 5 and intron 6)	Mild anterior cortical lens opacity (11 weeks); severe anterior cortical opacities (38 weeks)	Disorganized, irregularly shaped and swollen fiber cell and lens epithelium have vacuoles	Fiber cell disorganization	Dave et al. (2021)
*C57BL/6J*	Insertion of vector in exon 5	Mild nuclear cataract (P21), disrupted gradient refractive index (8 weeks) and increased resilience (8 weeks)	Misaligned meridional equatorial epithelial cells and lens fulcrum, disorganized fiber cells, disrupted suture apex centration and abnormal fiber cell membrane conductance	Abnormal nuclear fiber morphology and compaction	Cheng and Gong (2011), Cheng et al. (2013), Cheng et al. (2017), Cheng (2021), Cheng et al. (2021), Cheng et al. (2022)
*C57BL/6J*	Insertion of vector in exon 5	Smaller spherical lenses (2+ weeks) with reduced refractive power of the outer lens layers	Disorganized fiber cells, disturbed lens gradient index, and suture misalignment	Disrupted migration of fiber cells	Shi et al. (2012)
*C57BL/6J*	Insertion of vector in exon 5	Small lens with degraded optical quality (P21)	Decreased proliferation of lens epithelial cells, misaligned fiber cells with disturbed suture formation	Defective early patterning in cell differentiation contribute to later defects in patterning	Zhou and Shiels, (2018)

*Ephrin-A5* mutant genetic background	Knock-out/-in strategy	Phenotype (Age)	Cellular changes	Potential cause for cataracts	References
EphA2-R722Q *C57BL/6J*	CRISPR/Cas9 gene editing	No obvious cataracts (P21–12 months)	Longer/unequal posterior suture branches at P30	Patterning defects due to the disruption of cytoskeleton-associated protein expression	Zhou et al. (2021)
EphA2-del722 *C57BL/6J*	CRISPR/Cas9 gene editing	Translucent regions (P21–12 months)	Disrupted meridional epithelial-to-fiber cell alignment at the equator, deviated polar axis at the posterior pole with severe suture defects, misaligned hexagonal fiber cell radial columns, and retention of EphA2 in the cytoplasm	Patterning defects in the epithelial and fiber cells and disruption of cytoskeleton-associated protein expression	Zhou et al. (2021)

*Ephrin-A5* ^*−/−*^ genetic background	Knockout strategy	Phenotype (Age)	Cellular changes	Potential cause for cataracts	References
Mixed *129/Sv:C57BL/6*	Insertion of vector in exon	Progressive cortical cataracts with rupture of the posterior lens capsule (2 months)	Disorganized and rounded fiber cells	Loss of cell-cell adhesion due to loss of N-cadherin from the cytoplasmic membrane	Cooper et al. (2008)
Mixed *129/Sv:C57BL/6*	Insertion of vector in exon	Nuclear cataract (P21)	Presence of large vacuoles in the equatorial region, severe fiber cell degeneration, and lens rupture	Affected fiber cell packing during epithelial cell differentiation	Son et al. (2013)
Mixed *129/Sv:C57BL/6*	Insertion of vector in exon	Nuclear cataracts (2 months) followed by posterior capsule rupture	Disruption of N-cadherin-β-catenin complex that affects the interlocking protrusions causing cataract	Presence of membranous globules along the fiber cells disrupted interlocking protrusions in fiber cells	Biswas et al. (2016)
*C57BL/6J*	Insertion of vector in exon	Anterior polar cataracts (P21), mild decrease in maximum refractive index (8 weeks) and increased resilience (8 weeks)	Disruption of cell-cell adhesion (E-cadherin and β-catenin) in anterior epithelial cells leading to abnormal proliferation, disorganized suture apex centration and abnormal fiber cell membrane conductance	Aberrant EMT in anterior epithelial cells invading underlying fiber cells	Cheng and Gong (2011), Cheng et al. (2017), Cheng (2021), Cheng et al. (2021), Cheng et al. (2022)
*C57BL/6J*	Insertion of vector in exon	Small lens with degraded optical quality (P21)	Disturbed epithelial meridional pattern, radial fiber cell pattern, and suture disorganization	Defective early patterning in cell differentiation contributes to later defects in patterning	Zhou and Shiels, (2018)

*EphA2* ^*−/−*^ *ephrin-A5* ^*−/−*^ double knockout genetic background	Knockout strategy	Phenotype (Age)	Cellular changes	Potential cause for cataracts	References
*C57BL/6J*	Insertion of vector in exon	Mild nuclear cataract (P21) and anterior polar cataract (P21) (additive phenotype from each single knockout)	Misaligned meridional equatorial epithelial cells and disorganized fiber cells and aberrant proliferation of anterior epithelial cells	Fiber cell disruptions and aberrant EMT in anterior epithelial cells	Cheng et al. (2017)

### Function of EphA2 in Organizing Equatorial Epithelial Cells and Fiber Cells

The expression of EphA2 receptor proteins in the lens was identified at equatorial epithelial cell and fiber cell membranes and in fiber cell tips (Figure 3A) (Jun et al., 2009; Cheng and Gong, 2011; Cheng et al., 2017; Zhou and Shiels, 2018). The levels of EphA2 in normal mouse lenses were found to decline with age (Jun et al., 2009). The first report of the lens phenotype in *EphA2* ^*−/−*^ mice revealed cortical cataracts that progressed to whole cataracts and lens rupture with age (Table 2) (Jun et al., 2009). These KO animals were in the *FVB/NJ* genetic background, and EphA2 proteins were sequestered through gene trapping (Mitchell et al., 2001; Guo et al., 2006), leading to the formation of aggregates in these *EphA2* ^*−/−*^ lenses (Dave et al., 2021). A member of the heat shock protein family, Hsp25, was found to be significantly upregulated in these *EphA2* ^*−/−*^ lenses (Jun et al., 2009). The progressive and severe cataracts in these KO animals were hypothesized to be due to increased cellular stress and misfolding of proteins (Jun et al., 2009). The secretory gene trapping KO strategy involves the insertion of a secretory trapping vector between exon 5 and intron 6 of the *EphA2* gene, resulting in a truncated form of EphA2 without exons 6 through 17 (Mitchell et al., 2001). The partial translated EphA2 ectodomain is bound to a reporter protein, β-galactosidase, that traps the fusion protein in the cytoplasm and forms aggregates. The aggregates trigger a moderate unfolded-protein response (UPR) (Jun et al., 2009; Dave et al., 2021). A second gene trapping *EphA2* ^*−/−*^ mouse line in the mixed *129/SvJ:C57BL/6J* genetic background was also examined (Jun et al., 2009). This second gene trap mouse line had insertion of the gene trap in intron 1 resulting in truncated EphA2 protein with exon 1 fused to β-galactosidase (Naruse-Nakajima et al., 2001). The two gene trapping *EphA2* ^*−/−*^ mouse lines have similar cataract phenotypes (Jun et al., 2009). It is not clear whether UPR in these *EphA2* ^*−/−*^ lenses directly affects cataract phenotype and severity.

A recent report showed that when the exon 5/intron 6 gene-trapping *EphA2* ^*−/−*^ mice were backcrossed to the *C57BL/6J* genetic background, the KO lenses developed progressively more severe cortical cataracts, but the opacity did not progress to whole cataracts or lens rupture (Dave et al., 2021). Thus, it is likely that genetic modifiers in the *C57BL/6J* background affect cataract severity and phenotype. This notion is supported by previous studies showing that the severity of nuclear cataracts due to gap junction disruption is modulated by the *C57BL/6J* genetic background (Gong et al., 1998; Gong et al., 1999). Subsequent studies of another *EphA2* ^*−/−*^ mouse line, which utilized an exon deletion strategy, in the *C57BL/6J* genetic background, revealed mild nuclear cataracts (Figure 3C) in young mice and abnormal refractive properties (Cheng and Gong, 2011; Shi et al., 2012; Cheng et al., 2022). Consistent with data from the gene-trapping *EphA2* ^*−/−*^ lenses, there is disorganization of lens fiber cells due to the loss of EphA2 (Figure 3B) (Cheng and Gong, 2011; Shi et al., 2012; Cheng et al., 2013; Cheng et al., 2017; Cheng, 2021; Cheng et al., 2022). The genetic modifier(s) that affect cataract phenotype in the *C57BL/*6*J* background have yet to be identified in any KO mouse line.

Lens fiber cells, hexagonal in cross-section, allow low energy and tight packing conformations to minimize light scattering (Figure 2B) (Bassnett et al., 2011; Cheng et al., 2016). Hexagonal cell shape is initially established in equatorial epithelial cells as these differentiating cells organize into meridional rows (Bassnett et al., 2011). The mechanism for this remarkable morphogenesis relies on EphA2 signaling. EphA2 receptors are present at the cell membrane (Bassnett, 2009; Cheng et al., 2013; Cheng et al., 2017) of equatorial epithelial cells, differentiating and mature lens fibers as well as anterior fiber cell tips (Figure 3A) (Jun et al., 2009; Cheng et al., 2017; Zhou and Shiels, 2018). EphA2 recruits Src kinase to the vertices of hexagonal equatorial epithelial cells. Src is then activated by phosphorylation to recruit and activate cortactin to enrich actin at the cell vertices to establish and maintain hexagon cell shape (Figure 3B) (Cheng et al., 2013). Loss of EphA2 causes equatorial lens epithelial cells to have disrupted cell shape that leads to misaligned meridional rows, which in turn leads to disorganization of lens fiber cells (Cheng et al., 2013). In addition to abnormal cell shape, the characteristic undulating surface morphology and presence of protrusions and interdigitations in fiber cells were also disturbed in *EphA2* ^*−/−*^ mice (Shi et al., 2012; Cheng et al., 2022).

The avascular lens relies on a network of gap junction plaques, water channels, and active transport of sodium ions out of the lens to generate its own microcirculation current to bring in nutrients and remove waste (Mathias et al., 1981; Mathias et al., 1997). The loss of EphA2 alters connexin 50 (Cx50) localization to lens fiber cell membranes, presumably compromising gap junction plaque formation and cell-cell communication (Cheng et al., 2021). Loss of either ephrin-A5 or EphA2 also changes the localization of aquaporin 0, a protein that makes up water channels between cells (Cheng et al., 2021). Surprisingly, these defects do not affect cell-cell coupling in the lens, but disrupt the normal intracellular voltage and membrane conductance of lens fibers. This is the first evidence that these properties of lens fibers could be modulated and that Eph-ephrin signaling is involved in maintaining the ion homeostasis of fiber cells (Cheng et al., 2021).

*In vitro* studies have been used to understand the mechanism and effect of EphA2 mutations in cataract formation. In HEK293T and αTN4-1 cells, mutations in the SAM domain of EphA2 were found to induce instability, insolubility and dergradation of mutant proteins via a proteasome-dependent pathway (Park et al., 2012). In addition, transfection experiments using epithelial cell culture systems, such as Madin-Darby canine kidney (MDCK) and human colorectal adenocarcinoma (Caco-2) epithelial cells, revealed that SAM domain mutations of EphA2, namely p.T940I and p.D942fsXC7, affect the intercellular contacts due to destabilization of mutant EphA2 proteins (Dave et al., 2016). These two mutations were previously identified to cause severe total, nuclear or posterior polar congenital cataracts in humans (Zhang et al., 2009; Dave et al., 2013). Thus, changes in the EphA2 protein characteristics and conformation results in cataract formation.

### Eph-ephrin Signaling and Lens Biomechanics

Elongating fiber cell tips migrate along the apical surface of epithelial cells anteriorly and along the capsule posteriorly (Figure 2B). At the anterior and posterior poles, the fiber cell tips detach from the anterior epithelium or posterior capsule, respectively, and contact fiber cells from the opposing directions to form the Y-suture (Kuszak et al., 2004a; Kuszak et al., 2004b; Lovicu and Robinson, 2004). Examination of *EphA2* ^*−/−*^ and *ephrin-A5* ^*−/−*^ lenses revealed changes in the formation of Y-sutures at the anterior and posterior poles. In both *EphA2* ^*−/−*^ and *ephrin-A5* ^*−/−*^ lenses, the apex of the Y-suture is disorganized between concentric fiber cells layers (Cheng, 2021), and these KO lenses more often display additional, abnormal branching of the Y-suture (Zhou and Shiels, 2018; Cheng, 2021), with branching patterns similar to human lenses (Koretz et al., 1994; Kuszak et al., 2004b). Loss of either EphA2 or ephrin-A5 causes mouse lenses to become more spherical, possibly due to the suture patterning defect (Shi et al., 2012; Zhou and Shiels, 2018; Cheng, 2021). Unexpectedly, these suture defects did not change the stiffness of *EphA2* ^*−/−*^ and *ephrin-A5* ^*−/−*^ lenses, but these KO lenses were more resilient and recovered more fully after compressive load removal (Cheng, 2021). Increased resilience in KO lenses was due to a change in suture gap area under compression and recovery after load removal (Cheng, 2021). These data indicate that Eph-ephrin signaling influences suture formation, possibly by guiding the migration of lens fiber cell tips toward the anterior and posterior poles and determining the location of the suture apex. The change in resilience due to suture mispatterning suggests that the shape of the Y-suture constrains the elasticity of the lens (Cheng, 2021).

Mice containing an *EphA2* mutation (p.R722Q), which is homologus to the human *EPHA2* mutation (p.R721Q) associated with age-related cataracts (Jun et al., 2009), were generated using CRISPR/Cas9 technology (Zhou et al., 2021). The generation of mutant EphA2-R722Q mice also resulted in a separate off-target insertion-deletion mutant allele in exon 13 (EphA2-indel722). EphA2-R722Q mutant lenses (homozygous and heterozygous) were similar in size and transparency to control wild-type lenses, but EphA2-indel722 mutant lenses exhibited translucent regions, disrupted alignment of equatorial hexagonal epithelial and fiber cells, and polar axis shift of the fiber cell tips with severely disrupted suture pattern at the posterior pole (Zhou et al., 2021). There was no significant manifestation of cataract in young (3 weeks old) and old (12 months old) heterozygous and homozygous EphA2-R722Q and EphA2-indel722 mutant mice (Zhou et al., 2021). These results strengthen the hypothesis that EphA2 is required for the precise alignment of fiber cells at the equator and the formation of suture at the posterior pole.

Lifelong lens growth relies on the addition of new layers of lens fiber cells that surround previous generations of cells in concentric shells (Figure 2B). As the fiber cells mature, they are compacted and form a stiff lens nucleus, the center region of the lens (Al-Ghoul et al., 2001; Heys et al., 2004; Lovicu and Robinson, 2004). Recent studies have shown that the compacted lens nucleus is correlated with areas of high refractive index in mouse lenses (Cheng et al., 2019; Cheng et al., 2022). It has long been hypothesized that increased stiffness of the lens nucleus with age increases overall lens stiffness and contributes to the development of presbyopia (Heys et al., 2004; Weeber et al., 2007). We recently examined the morphometric properties of control, *EphA2* ^*−/−*^, and *ephrin-A5* ^*−/−*^ lenses. Unexpectedly, we found that *EphA2* ^*−/−*^ lenses had smaller and softer lens nuclei, which correlated with decreased gradient refractive index (Cheng et al., 2022). Loss of EphA2 affects mature and perinuclear lens fiber cell morphology leading to abnormal tongue-and-groove interdigitations and loss of normal interlocking protrusions (Cheng et al., 2022). Interestingly, the change in lens nucleus size and stiffness in *EphA2* ^*−/−*^ lenses does not affect overall whole lens stiffness (Cheng et al., 2022).

### Binding Partners of EphA2 and Ephrin-A5 and Crosstalk With Other Signaling Pathways

Some tissue and cell culture studies have shown that EphA2 and ephrin-A5 interact with each other to regulate cellular functions, like cell migration (Park et al., 2012), tumorigenicity (Li et al., 2009), and invasive properties of breast cancer (Shaw et al., 2014). Built on these findings, some groups have suggested that EphA2 and ephrin-A5 could be binding partners in the lens (Cooper et al., 2008; Shi et al., 2012; Son et al., 2013). In lens epithelial cells, differences in the epithelial cell phenotype between *EphA2* ^*−/−*^ and *ephrin-A5* ^*−/−*^ lenses suggested that the two proteins function independently of each other. Genetic studies of double KO *EphA2* ^*−/−*^ *ephrin-A5* ^*−/−*^ lenses revealed that anterior and equatorial lens epithelial cell phenotypes were additive in the double mutant mice, indicating that EphA2 and ephrin-A5 are not receptor-ligand pair in lens epithelial cells (Cheng et al., 2017). Ephrin-A5 maintains the quiescence of anterior lens epithelial cells, while EphA2 regulates equatorial epithelial cell shape and organization (Cheng and Gong, 2011; Cheng et al., 2013). Studies of lens fiber cell tips and Y-suture formation suggest that loss of EphA2 or ephrin-A5 lead to similar defects in fiber cells at that specific location, indicating possibly that EphA2 and ephrin-A5 interact in this region of the lens (Zhou and Shiels, 2018; Cheng, 2021). This hypothesis is supported by EphA2 and ephrin-A5 immunostaining signals being present at the fiber cell tips near the lens suture (Cheng and Gong, 2011; Cheng et al., 2017). Most cells express a complement of Ephs and ephrins, and thus, in the lens, compensatory mechanisms for the loss of one receptor or ligand could lead to upregulation or downregulation of other receptors and/or ligands in the lens epithelial and fiber cells, which has yet to be investigated.

EphA2 signals through the Src and cortactin pathway to influence actin cytoskeleton in equatorial lens epithelial cells to regulate hexagon cell shape and cell organization (Cheng et al., 2013), similar to interactions previously shown in other tissues between EphA2 and Src (Baldwin et al., 2006; Parri et al., 2007; Faoro et al., 2010). A recent study has confirmed complex formation between EphA2 and Src kinase thereby supporting their direct downstream interaction (Zhou et al., 2021). The development and maintenance of the lens’ unique structure are determined by growth factors and signaling pathways (Beebe et al., 1987; Belecky-Adams et al., 1997; Faber et al., 2002; Lovicu and McAvoy, 2005). Fibroblast growth factors (FGF), another group of RTK, have been identified to antagonistically interact with ephrin-B ligands (Pasquale, 2008), resulting in asymmetric cell division and cell fate determination in *Ciona* embryos (Picco et al., 2007) and in *Xenopus* eye field formation (Moore et al., 2004). Discs large-1 (Dlg-1), a PDZ protein (Rivera et al., 2009), and FGF (Wang et al., 2010) were found to be involved in lens fiber cell differentiation. Loss of Dlg-1 (Rivera et al., 2009) in mouse lens led to defective fiber cell patterning and cell-cell adhesion. Dlg-1 interacts with EphA2 to influence FGF signaling and regulate lens fiber cells during differentiation and structural maintenance (Lee et al., 2016), suggesting crosstalk between Eph-ephrin signaling, FGF signaling and adherens junctions. In addition, loss of ephrin-A5 caused abnormal association of N-cadherin and β-catenin in lens fibers of mixed background KO mice (Cooper et al., 2008) as well as disrupted E-cadherin and β-catenin staining in anterior epithelial cells of *C57BL/6J* background KO anterior epithelial cells (Cheng and Gong, 2011). These data further indicate the Eph-ephrin signaling is needed for normal cell-cell adhesion and adherens junctions in the lens.

### Future Directions and Therapeutic Opportunities

Eph-ephrin signaling has a wide range of roles during development (e.g., angiogenesis (Salvucci and Tosato, 2012), tissue patterning (Henkemeyer et al., 1994) and neural development (Holmberg et al., 2000)), as well as in the physiology of adult tissues (e.g., insulin secretion (Konstantinova et al., 2007; Jain et al., 2013), bone homeostasis (Zhao et al., 2006)) and in diseases including cancer (Clevers and Batlle, 2006; Guo et al., 2006) and neurological disorders (Fu et al., 2014)). The receptors and ligands can play both inhibitor and activator roles through canonical ligand-mediated or non-canonical ligand-independent pathways. From studies using mice to understand the roles of Eph-ephrin signaling in cataractogenesis, the strain background can greatly influence the phenotype and severity and progression of cataracts (Cheng and Gong, 2011; Shi et al., 2012; Cheng et al., 2013; Son et al., 2013; Biswas et al., 2016; Zhou and Shiels, 2018). This has complicated our interpretation of the lens phenotypes and the ability to compare data across different studies. However, the variable cataract phenotypes in mice recapitulate the large variety of human cataracts and suggests the possibility that multiple genetic modifiers modulate cataract severity and progression. Studies to identify genetic modifiers in mice could help unravel cataractogenesis mechanisms in human patients.

While Ephs and ephrins have been studied extensively in other tissues, we are still working toward mapping the spatiotemporal expression patterns of relevant Ephs and ephrins in the lens and determining their direct binding partners and the downstream signaling pathways. In-depth knowledge of the receptors, ligands, their interactions with each other and with other downstream effectors is necessary to understand and formulate therapeutic strategies. Recently, studies have shown the role of Ephs and ephrins in age-related diseases, like Alzheimer’s (Cisse et al., 2011) and Parkinson’s disease (Jing et al., 2012). Inhibition of specific Ephs can promote the regeneration of damaged neural networks (Fabes et al., 2007; Goldshmit et al., 2011; Spanevello et al., 2013) and control tumor microenvironment (Miao and Wang, 2012; Salvucci and Tosato, 2012), and their activation can affect vascular development, cardioprotection and heart tissue maintenance (Stephen et al., 2007; Genet et al., 2012; Goichberg et al., 2013). Recombinant extracellular domains, antibodies, peptides, small molecule agonists and antagonists, antisense oligonucleotides, or siRNAs are some of the therapeutic molecules that can be used to target the Eph-ephrin signaling to either inhibit or activate the signaling pathway to treat various diseases (Barquilla and Pasquale, 2015). We hope that a better understanding of the universe of Ephs and ephrins in the lens and the mechanisms for current therapeutic strategies can be translated to future anti-aging treatment for ocular diseases, like presbyopia and cataracts.
